# The evolution of methods for the capture of human movement leading to markerless motion capture for biomechanical applications

**DOI:** 10.1186/1743-0003-3-6

**Published:** 2006-03-15

**Authors:** Lars Mündermann, Stefano Corazza, Thomas P Andriacchi

**Affiliations:** 1 Department of Mechanical Engineering, Stanford University, Stanford, CA, USA; 2 Bone and Joint Research Center, VA Palo Alto, Palo Alto, CA, USA; 3 Department of Orthopedics, Stanford University, Stanford, CA, USA

## Abstract

Over the centuries the evolution of methods for the capture of human movement has been motivated by the need for new information on the characteristics of normal and pathological human movement. This study was motivated in part by the need of new clinical approaches for the treatment and prevention of diseases that are influenced by subtle changes in the patterns movement. These clinical approaches require new methods to measure accurately patterns of locomotion without the risk of artificial stimulus producing unwanted artifacts that could mask the natural patterns of motion. Most common methods for accurate capture of three-dimensional human movement require a laboratory environment and the attachment of markers or fixtures to the body's segments. These laboratory conditions can cause unknown experimental artifacts. Thus, our understanding of normal and pathological human movement would be enhanced by a method that allows the capture of human movement without the constraint of markers or fixtures placed on the body. In this paper, the need for markerless human motion capture methods is discussed and the advancement of markerless approaches is considered in view of accurate capture of three-dimensional human movement for biomechanical applications. The role of choosing appropriate technical equipment and algorithms for accurate markerless motion capture is critical. The implementation of this new methodology offers the promise for simple, time-efficient, and potentially more meaningful assessments of human movement in research and clinical practice. The feasibility of accurately and precisely measuring 3D human body kinematics for the lower limbs using a markerless motion capture system on the basis of visual hulls is demonstrated.

## Introduction

Over the last several centuries our understanding of human locomotion has been a function of the methods to capture human movement that were available at the time. In many cases the expanded need for enhancing our understanding of normal and pathological human movement drove the introduction of new methods to capture human movement.

### Historical examples

A look at the history of the study of human locomotion provides some interesting examples of contemporary problems driving the development of new methods for the capture and analysis of human movement. For example, the Weber brothers (1836) reported one of the first quantitative studies of the temporal and distance parameters during human locomotion [1]. Their work established a model for subsequent quantitative studies of human locomotion. The works of two contemporaries, Marey (1873) and Muybridge (1878), were among the first to quantify patterns of human movement using photographic techniques [2,3]. Also during that time period, Wilhelm Braune (an anatomist) and Otto Fisher (a mathematician) reported measurements of body segment movements to calculate joint forces and energy expenditures using Newtonian mechanics [4]. Interestingly, their work was motivated by military applications related to improving the efficiency of troop movement.

During the 1950s there was a need for an improved understanding of locomotion for the treatment of World War II veterans. The classic work at the University of California [5,6] provided a tremendous resource of knowledge related to the mechanics of human movement. The work at the University of California formed the basis for many of the fundamental techniques currently used for the study of human locomotion. More recently, instrumentation and computer technologies have provided new opportunities for the advancement of the study of human locomotion. The limitations with respect to automated motion capture as well as measurement reduction no longer exist. New methodology has made it feasible to extend the application of kinetic analysis to clinical problems.

### Current state of the art

As discussed the expanded need for improved knowledge of locomotion drove the invention of new methods of observation. At present, the most common methods for accurate capture of three-dimensional human movement require a laboratory environment and the attachment of markers, fixtures or sensors to the body segments. These laboratory conditions can cause unknown experimental artifacts.

Currently, one of the primary technical factors limiting the advancement of the study of human movement is the measurement of skeletal movement from markers or sensors placed on the skin. The movement of the markers is typically used to infer the underlying relative movement between two adjacent segments (e.g. knee joint) with the goal of precisely defining the movement of the joint. Skin movement relative to the underlying bone is a primary factor limiting the resolution of detailed joint movement using skin-based systems [7-11].

Skeletal movement can also be measured directly using alternative approaches to a skin-based marker system. These approaches include stereoradiography [12], bone pins [9,13], external fixation devices [10] or single plane fluoroscopic techniques [14,15]. While these methods provide direct measurement of skeletal movement, they are invasive or expose the test subject to radiation. More recently, real-time magnetic resonance imaging (MRI) using open-access MRI provide non-invasive and harmless *in vivo *measurement of bones, ligaments, muscle, etc. [16]. However, all these methods also impede natural patterns of movements and care must be taken when attempting to extrapolate these types of measurements to natural patterns of locomotion. With skin-based marker systems, in most cases, only large motions such as flexion-extension have acceptable error limits. Cappozzo et al. [17] have examined five subjects with external fixator devices and compared the estimates of bone location and orientation between coordinate systems embedded in the bone and coordinate systems determined from skin-based marker systems for walking, cycling and flexion-extension activities. Comparisons of bone orientation from true bone embedded markers versus clusters of three skin-based markers indicate a worst-case root mean square artifact of 7°.

The most frequently used method for measuring human movement involves placing markers or fixtures on the skin's surface of the segment being analyzed [18]. The vast majority of current analysis techniques model the limb segment as a rigid body, then apply various estimation algorithms to obtain an optimal estimate of the rigid body motion. One such rigid body model formulation is given by Spoor and Veldpas [19]; they have described a rigid body model technique using a minimum mean square error approach that lessens the effect of deformation between any two time steps. This assumption limits the scope of application for this method, since markers placed directly on skin will experience non-rigid body movement. Lu and O'Connor [20] expanded the rigid body model approach; rather than seeking the optimal rigid body transformation on each segment individually, multiple, constrained rigid body transforms are sought, modeling the hip, knee, and ankle as ball and socket joints. The difficulty with this approach is modeling the joints as ball and sockets where all joint translations are treated as artifact, which is clearly a limitation for knee motion. Lucchetti et al. [21] presented an entirely different approach, using artifact assessment exercise to determine the correlation between flexion-extension angles and apparent skin marker artifact trajectories. A limitation of this approach is the assumption that the skin motion during the quasi-static artifact assessment movements is the same as during dynamic activities.

A recently described [22,23] point cluster technique (PCT) employs an overabundance of markers (a cluster) placed on each segment to minimize the effects of skin movement artifact. The basic PCT [24] can be extended to minimize skin movement artifact by optimal weighting of the markers according to their degree of deformation. Another extension of the basic PCT corrects for error induced by segment deformation associated with skin marker movement relative to the underlying bone. This is accomplished by extending the transformation equations to the general deformation case, modeling the deformation by an activity-dependent function, and smoothing the deformation over a specified interval to the functional form. A limitation of this approach is the time-consuming placement of additional markers.

In addition to skin movement artifact, many of the previously described methods can introduce an artificial stimulus to the neurosensory system while measuring human movement yielding motion patterns that do not reflect natural patterns of movement. For example, even walking on a treadmill can produce changes in the stride length-walking speed relationships [25]. Insertion of bone pins, the strapping of tight fixtures around limb segments or constraints to normal movement patterns (such as required for fluoroscopic or other radiographic imaging measurements) can introduce artifacts into the observation of human movement due to local anesthesia and/or interference with musculoskeletal structures. In some cases, these artifacts can lead to incorrect interpretations of movement data.

The potential for measurement-induced artifact is particularly relevant to studies where subtle gait changes are associated with pathology. For example, the success of newer methods for the treatment and prevention of diseases such as osteoarthritis [26] is influenced by subtle changes in the patterns of locomotion. Thus, the ability to accurately measure patterns of locomotion without the risk of an artificial stimulus producing unwanted artifacts that could mask the natural patterns of motion is an important need for emerging health care applications.

Ideally, the measurement system/protocol should be neither invasive nor harmful and only minimally encumber the subject. Furthermore, it should allow measuring subjects in their natural environment such as their work place, home, or on sport fields and be capable of measuring natural activities/motion over a sufficiently large field of view. The purpose of this paper is to examine the development of markerless methods for providing accurate representation of three-dimensional joint mechanics and addressing emerging needs for a better understanding of the biomechanics of normal and pathological motion. The terms markerless and marker-free are used interchangeable for motion capture system without markers. In this review we will use the term markerless motion capture.

### Markerless methods for human motion capture

Motion capture is an important method for studies in biomechanics and has traditionally been used for the diagnosis of the patho-mechanics related to musculoskeletal diseases [27,28]. Recently it has also been used in the development and evaluation of rehabilitative treatments and preventive interventions for musculoskeletal diseases [29]. Although motion analysis has been recognized as clinically useful, the routine clinical use of gait analysis has seen very limited growth. The issue of its clinical value is related to many factors, including the applicability of existing technology to addressing clinical problems and the length of time and costs required for data collection, processing and interpretation [30]. A next critical advancement in human motion capture is the development of a non-invasive and markerless system. A technique for human body kinematics estimation that does not require markers or fixtures placed on the body would greatly expand the applicability of human motion capture. Eliminating the need for markers would also considerably reduce patient preparatory time and enable simple, time-efficient, and potentially more meaningful assessments of human movement in research and clinical practice. To date, markerless methods are not widely available because the accurate capture of human movement without markers is technically challenging yet recent technical developments in computer vision provide the potential for markerless human motion capture for biomechanical and clinical applications.

One of the challenges for a markerless system is the acquisition and representation of human movement. Systems are typically divided into two categories, namely active and passive vision systems. Active systems emit light-information in the visible or infrared light spectrum in the form of laser light, light patterns or modulated light pulses, while passive systems rely purely on capturing images. In general, active systems such as laser scanners, structured light systems and time-of-flight sensors provide very accurate 3D measurements, but require a controlled laboratory environment and often are limited to static measurements. For example, a full body laser scan typically takes several seconds to capture the surface of a human body. Therefore, the main focus on the development of vision systems for markerless motion capture currently lies on employing passive systems. Passive systems are advantageous as they only rely on capturing images and thus provide an ideal framework for capturing subjects in their natural environment.

The development of markerless motion capture systems originated from the fields of computer vision and machine learning, where the analysis of human actions by a computer is gaining increasing interest. Potential applications of human motion capture are the driving force of system development, and the major application areas are: smart surveillance, identification, control, perceptual interface, character animation, virtual reality, view interpolation, and motion analysis [31,32]. Over the past two decades, the field of registering human body motion using computer vision has grown substantially, and a great variety of vision-based systems have been proposed for tracking human motion. These systems vary in the number of cameras used (camera configuration), the representation of captured data, types of algorithms, use of various models, and the application to specific body regions and whole body. Employed configurations typically range from using a single camera [33-35] to multiple cameras [36-40].

An even greater variety of algorithms has been proposed for estimating human motion including constraint propagation [41], optical flow [42,43], medial axis transformation [44], stochastic propagation [45], search space decomposition based on cues [36], statistical models of background and foreground [46], silhouette contours [47], annealed particle filtering [48], silhouette based techniques [49,50], shape-encoded particle propagation [51], and fuzzy clustering process [52]. These algorithms typically derive features either directly in the single or multiple 2D image planes [42,45] or, in the case of multiple cameras, at times utilize a 3D representation [36,50] for estimating human body kinematics, and are often classified into model-based and model-free approaches. The majority of approaches is model-based in which an a priori model with relevant anatomic and kinematic information is tracked or matched to 2D image planes or 3D representations. Different model types have been proposed including stick-figure [35], cylinders [33], super-quadrics [36], and CAD model [43]. Model-free approaches attempt to capture skeleton features in the absence of an a priori model. These include the representation of motion in form of simple bounding boxes [53] or stick-figure through medial axis transformation [44], and the use of Isomaps [54] and Laplacian Eigenmaps [55] for transforming a 3D representation into a pose-invariant graph for extracting kinematics.

Several surveys concerned with computer-vision approaches have been published in recent years, each classifying existing methods into different categories [31,32,56-58]. For instance, Moeslund et al. [31] reviewed more than 130 human motion capture papers published between 1980 and 2000 and categorized motion capture approaches by the stages necessary to solve the general problem of motion capture. Wang et. al [32] provided a similar survey of human motion capture approaches in the field of computer vision ranging mainly from 1997 to 2001 with a greater emphasize on categorizing the framework of human motion analysis in low-level vision, intermediate-level vision, and high-level vision systems.

While many existing computer vision approaches offer a great potential for markerless motion capture for biomechanical applications, these approaches have not been developed or tested for this applications. To date, qualitative tests and visual inspections are most frequently used for assessing approaches introduced in the field of computer vision and machine learning. Evaluating existing approaches within a framework focused on addressing biomechanical applications is critical. The majority of research on human motion capture in the field of computer vision and machine learning has concentrated on tracking, estimation and recognition of human motion for surveillance purposes. Moreover, much of the work reported in the literature on the above has been developed for the use of a single camera. Single image stream based methods suffer from poor performance for accurate movement analysis due to the severe ill-posed nature of motion recovery. Furthermore, simplistic or generic models of a human body with either fewer joints or reduced number of degrees of freedom are often utilized for enhancing computational performance. For instance, existing methods for gait-based human identification in surveillance applications use mostly 2D appearance models and measurements such as height, extracted from the side view. Generic models typically lack accurate joint information and thus lack accuracy for accurate movement analysis. However, biomechanical and, in particular, clinical applications typically require knowledge of detailed and accurate representation of 3D joint mechanics. Some of the most challenging issues in whole-body movement capture are due to the complexity and variability of the appearance of the human body, the nonlinear and non-rigid nature of human motion, a lack of sufficient image cues about 3D body pose, including self-occlusion as well as the presence of other occluding objects, and exploitation of multiple image streams. Human body self-occlusion is a major cause of ambiguities in body part tracking using a single camera. The self-occlusion problem is addressed when multiple cameras are used, since the appearance of a human body from multiple viewpoints is available.

Approaches from the field of computer vision have previously been explored for biomechanical applications. These include the use of a model-based simulated annealing approach for improving posture prediction from marker positions [59] and marker-free systems for the estimation of joint centers [60], tracking of lower limb segments [61], analysis of movement disabilities [47,52], and estimation of working postures [62]. In particular, Persson [61] proposed a marker-free method for tracking the human lower limb segments. Only movement in the sagittal plane was considered. Pinzke and Kopp [62] tested the usability of different markerless approaches for automatic tracking and assessing identifying and evaluating potentially harmful working postures from video film. Legrand et al. [47] proposed a system composed of one camera. The human boundary was extracted in each image and a two-dimensional model of the human body, based on tapered super-quadrics, was matched. Marzani et al. [52] extended this approach to a system consisting of three cameras. A 3D model based on a set of articulated 2D super-quadrics, each of them describing a part of the human body, was positioned by a fuzzy clustering process.

These studies demonstrate the applicability of techniques in computer vision for automatic human movement analysis, but the approaches were not validated against marker-based data. To date, the detailed analysis of 3D joint kinematics through a markerless system is still lacking. Quantitative measurements of movement and continuous tracking of humans using multiple image streams is crucial for 3D gait studies. A markerless motion capture system based on visual hulls from multiple image streams and the use of detailed subject-specific 3D articulated models with soft joint constraints is demonstrated in the following section. To critically analyze the effectiveness of markerless motion capture in the biomechanical/clinical environment, we quantitatively compared data obtained from this new system with data obtained from marker-based motion capture.

### Markerless human movement analysis through visual hull and articulated ICP

The overall goal of our research is to develop a markerless system using multiple optical sensors that will efficiently and accurately provide 3D measurements of human movement for application in clinical practice. Our approach employs an articulated iterative closest point (ICP) algorithm with soft joint constraints [63] for tracking human body segments in visual hull sequences (a standard 3D representation of dynamic sequences from multiple images). The soft joint constraints approach extends previous approaches [42,50] for tracking articulated models that enforced hard constraints on the joints of the articulated body. Small movements at the joint are allowed and penalized in least-squares terms. As a result a more anatomically correct matching suitable for biomechanical applications is obtained with an objective function that can be optimized in an efficient and straightforward manner.

The articulated ICP algorithm is a generalization of the standard ICP algorithm [64,65] to articulated models. The objective is to track an articulated model in a sequence of visual hulls. The articulated model *M *is represented as a discrete sampling of points *p*_*1*_, ..., *p*_*P *_on the surface, a set of rigid segments *s*_*1*_, ..., *s*_*S*_, and a set of joints *q*_*1*_, ..., *q*_*Q *_connecting the segments. Each visual hull is represented as a set of points *V *= *v*_*1*_, ..., *v*_*N*_, which describes the appearance of the person at that time. For each frame of the sequence, an alignment *T *is computed, which brings the surfaces of *M *and *V *into correspondence, while respecting the model joints *q*. The alignment *T *consists of a set of rigid transformations *T*_*j*_, one for each rigid part *s*_*j*_. Similar to ICP, this algorithm iterates between two steps. In the first step, each point *p*_*i *_on the model is associated to its nearest neighbor *v*_*s*(*i*) _among the visual hull points *V*, where *s(i) *defines the mapping from the index of a surface point *p*_*i *_to its rigid part index. In the second step, given a set of corresponding pairs (*p*_*i*_, *v*_*s*(*i*)_), a set of transformations *T *is computed, which brings them into alignment. The second step is defined by an objective function of the transformation variables given as *F(T) *= *H(T) *+ *G(T)*. The term *H(T) *ensures that corresponding points (found in the first step) are aligned.



The transformation *T*_*j *_of each rigid part *s*_*j *_is parameterized by a 3 × 1 translation vector *t*_*j *_and a 3 × 1 twist coordinates vector *r*_*j *_(twists are standard representations of rotation [66]), and *R(r*_*s*(*i*)_*) *denotes the rotation matrix induced by the twist parameters *r*_*s*(*i*)_. The term *G(T) *ensures that joints are approximately preserved, where each joint *q*_*i*,*j *_can be viewed as a point belonging to parts *s*_*i *_and *s*_*j *_simultaneously. The transformations *T*_*i *_and *T*_*j *_are forced to predict the joint consistently.



Decreasing the value of *w*_*G *_allows greater movement at the joint, which potentially improves the matching of body segments to the visual hull. The center of the predicted joint locations (belonging to adjacent segments) provides an accurate approximation of the functional joint center. As a result, the underlying kinematic model can be refined and a more anatomically correct matching is obtained.

The algorithm was evaluated in a theoretical and experimental environment [67,68]. The accuracy of human body kinematics was evaluated by tracking articulated models in visual hull sequences. Most favorable camera arrangements for a 3 × 1.5 × 2 m viewing volume were used [69]. This viewing volume is sufficiently large enough to capture an entire gait cycle. The settings w_H _= 1, w_G _= 5000 (Equations 1 and 2) were used to underscore the relative importance of the joints. The theoretical analysis was conducted in a virtual environment using a realistic human 3D model. The virtual environment permitted the evaluation of the quality of visual hulls on extracting kinematics while excluding errors due to camera calibration and fore-/background separation. To simulate a human form walking, 120 poses were created using Poser (Curious Labs, CA) mimicking one gait cycle. The poses of the human form consisted of 3D surfaces and had an average volume of 68.01 ± 0.06 liters. Visual hulls of different quality using 4, 8, 16, 32 and 64 cameras with a resolution of 640 × 480 pixels and an 80-degree horizontal view were constructed of the Poser sequence. In the experimental environment, full body movement was captured using a marker-based and a markerless motion capture system simultaneously. The marker-based system consisted of an eight-Qualisys camera optoelectronic system monitoring 3D marker positions for the hip, knees and ankles at 120 fps. The markerless motion capture system consisted of eight Basler CCD color cameras (656 × 494 pixels; 80-degree horizontal view) synchronously capturing images at 75 fps. Internal and external camera parameters and a common global frame of reference were obtained through offline calibration. Images from all cameras were streamed in their uncompressed form to several computers during acquisition.

**Figure 1:**
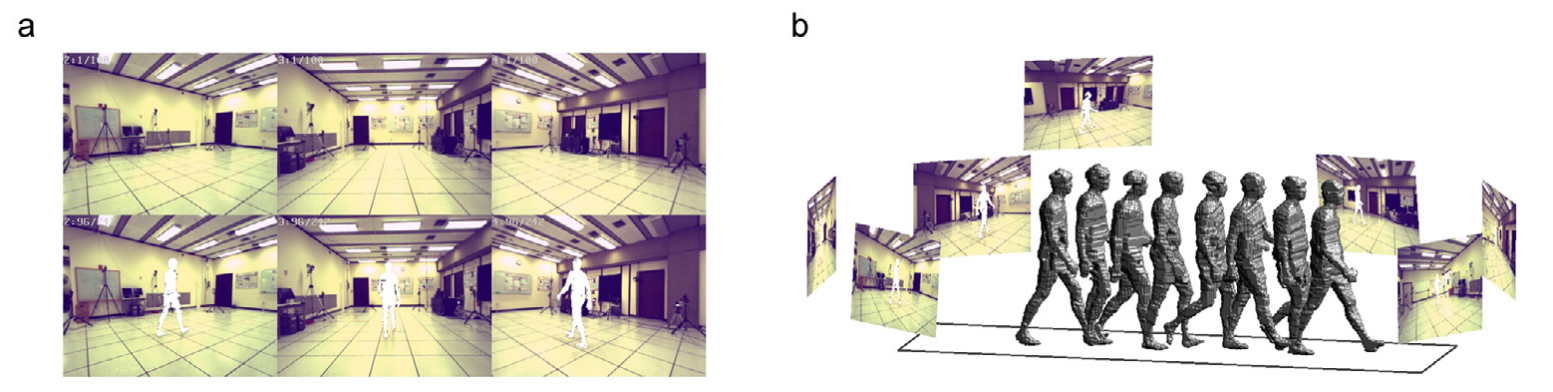
(a) Selected background images (top) and separated subject data (bottom). (b) Camera configuration, video sequences with separated subject data, and selected visual hulls.

**Figure 2:**
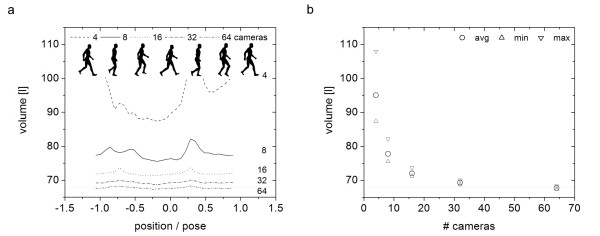
(a) Volume values of visual hulls as a function of position and pose in the viewing volume. (b) Average, min and max volume values across the viewing volume as a function of number of cameras. The dotted line indicates the human form's volume.

The subject was separated from the background in the image sequence of all cameras using intensity and color thresholding [70] compared to background images (Figure 1). The 3D representation was achieved through visual hull construction from multiple 2D camera views [71-73]. Visual hulls were created with voxel edges of λ = 10 mm, which is sufficiently small enough for these camera configurations [74]. The number of cameras used for visual hull construction greatly affects the accuracy of visual hulls [69]. The accuracy of visual hulls also depends on the human subject's position and pose within an observed viewing volume [69]. Simultaneous changes in position and pose result in decreased accuracy of visual hull construction (Figure 2). Increasing the number of cameras leads to decreased variations across the viewing volume and a better approximation of the true volume value.

**Figure 3:**
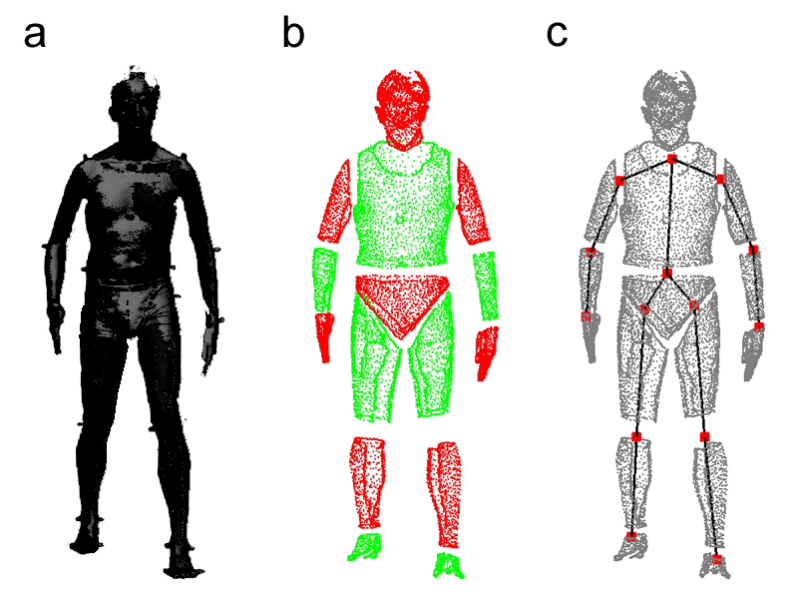
(a) Laser scan. (b) Body segments. (c) Joint centers.

A subject-specific 3D articulated model was tracked in the 3D representations constructed from the image sequences. An articulated model is typically derived from a morphological description of the human body's anatomy plus a set of information regarding the kinematic chain and joint centers. The morphological information of the human body can be a general approximation (cylinders, super-quadrics, etc.) or an estimation of the actual subject's outer surface. Ideally, an articulated model is subject-specific and created from a direct measurement of the subject's outer surface. The kinematic chain underneath an anatomic model can be manually set or estimated through either functional [49,75] or anthropometric methods [76,77]. The more complex the kinematic description of the body the more information can be obtained from the 3D representation matched by the model. While in marker-based systems the anatomic reference frame of a segment is acquired from anatomical landmarks tracked consistently through the motion path, in the markerless system the anatomical reference frames are defined by the model joint centers and reference pose. During the tracking process, the reference frames remain rigidly attached to their appropriate model anatomic segment, thus describing the estimated position and orientation in the subject's anatomic segments. In this study, an articulated body was created from a detailed full body laser scan with markers affixed to the subject's joints (Figure 3). The articulated body consisted at least of 15 body segments (head, trunk, pelvis, and left and right arm, forearm, hand, thigh, shank and foot) and 14 joints connecting these segments.

**Figure 4:**
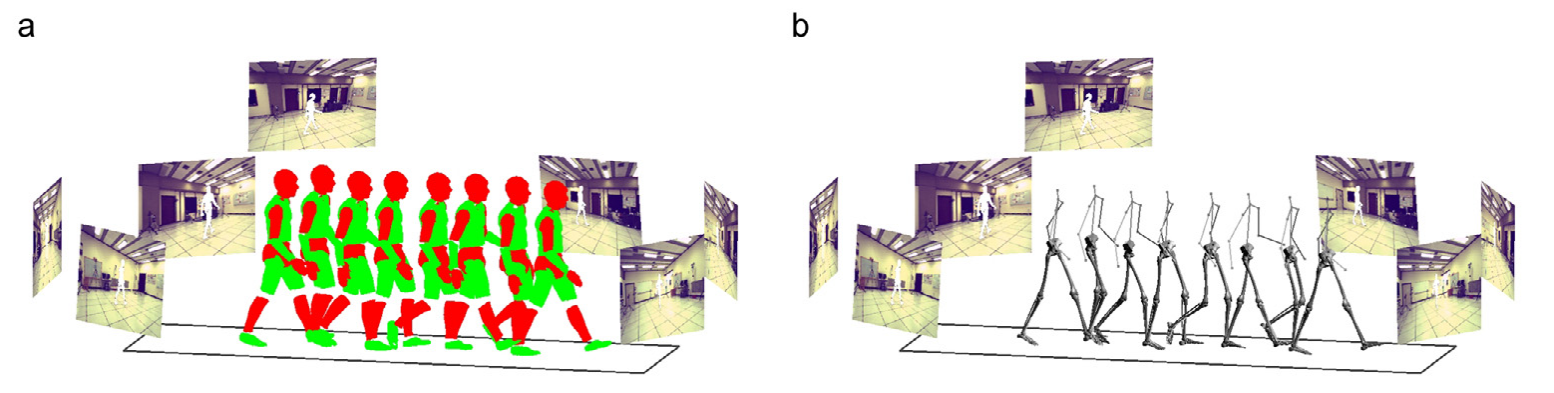
Articulated body matched to visual hulls. (a) Human body segments. (b) Kinematic chain.

**Figure 5:**
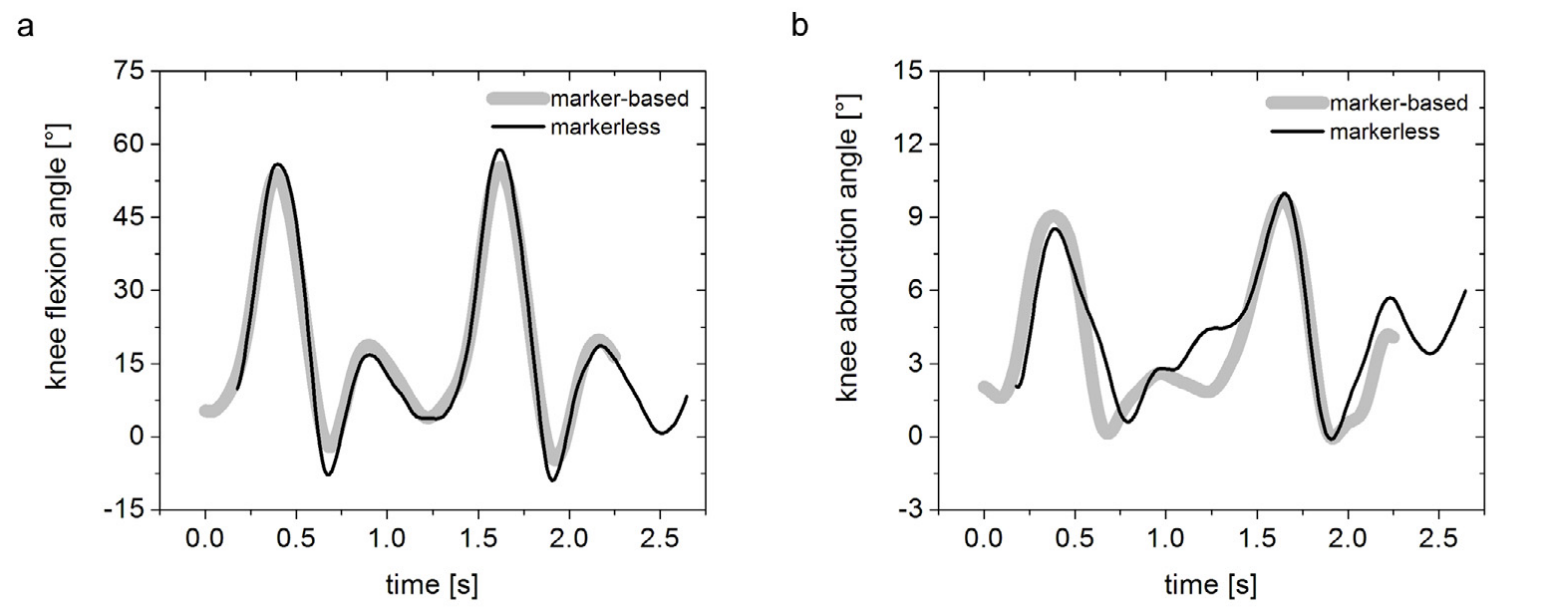
Motion graphs for (a) knee flexion and (b) knee abduction angles (gray = marker-based; black = markerless).

The subject's pose was roughly matched to the first frame in the motion sequence and subsequently tracked automatically over the gait cycle (Figure 4). Joint center locations were extracted for all joints and joint centers of adjacent segments were used to define segment coordinate axes. Joint angles for the lower limbs for the sagittal and frontal planes were calculated as angles between corresponding axes of neighboring segments projected into the corresponding planes. Accuracy of human body kinematics was calculated as the average deviation of the deviation of joint angles derived from visual hulls compared to joint angles derived from the theoretical sequence and marker-based system over the gait cycle, respectively. The joint angles (sagittal and frontal plane) for the knee calculated as angles between corresponding axes of neighboring segments are used as preliminary basis of comparison between the marker-based and markerless systems (Figure 5). The accuracy of sagittal and frontal plane knee joint angles calculated from experiments was within the scope of the accuracy estimated from the theoretical calculations (accuracy_experimental_: 2.3 ± 1.0° (sagittal); 1.6 ± 0.9° (frontal); accuracy_theoretical_: 2.1 ± 0.9° (sagittal); 0.4 ± 0.7° (frontal); [67,68]). A similar method, with different model matching formulation and limited to hard joint constraints, was recently explored by the authors [78]. This method utilized simulated annealing and exponential maps to extract subject's kinematics, and resulted in comparable accuracy.

This markerless system was recently used to investigate the role of trunk movement in reducing medial compartment load [79]. Conventional marker-based motion capture methods are not well suited to study whole body movement since they require a large number of markers placed all over the body. Subjects performed walking trials at a self-selected normal speed in their own low top, comfortable walking shoes with a) normal and b) increased medio-lateral trunk motion. On average, subjects increased their medio-lateral trunk sway by 7.9 ± 4.5° (P = 0.002) resulting in an average reduction of the first peak knee adduction moment of 68.1 ± 16.5% (P < 0.001). Subjects with greater increase in medio-lateral trunk sway experienced greater reductions in the first peak knee adduction moment. The magnitude of reductions in the first peak knee adduction moments were in some cases substantially greater than for conventional interventions including high tibial osteotomy or footwear interventions. The trunk movement assessed was similar to the natural gait compensation adopted by patients with knee OA such as Trendelenburg gait supporting previous findings [80,81] that the load distribution between the medial and lateral compartments at the knee during walking is critical. These results demonstrate that introducing a markerless motion capture system into clinical practice will provide meaningful assessments.

## Discussion

The development of markerless motion capture methods is motivated by the need to address contemporary needs to understand normal and pathological human movement without the encumbrance of markers or fixtures placed on the subject, while achieving the quantitative accuracy of marker based systems. Markerless motion capture has been widely used for a range of applications in the surveillance, film and game industries. However, the biomechanical, medical, and sports applications of markerless capture have been limited by the accuracy of current methods for markerless motions capture.

Previous experience has demonstrated that minor changes in patterns of locomotion can have a profound impact on the outcome of treatment or progression of musculoskeletal pathology. The ability to address emerging clinical questions on problems that influence normal patterns of locomotion requires new methods that would limit the risk of producing artifact due to markers or the constraints of the testing methods. For example, the constraints of the laboratory environment as well as the markers placed on the subjects can mask subtle but important changes to the patterns of locomotion. It has been shown that the mechanics of walking was changed in patients with anterior cruciate ligament deficiency of the knee [26,82]; functional loading influenced the outcome of high tibial osteotomy [83]; functional performance of patients with total knee replacement was influenced by the design of the implant [84], and the mechanics of walking influenced the disease severity of osteoarthritis of the knee [26,29,80,85]. It should be noted that each of the clinical examples referenced above were associated with subtle but important changes to the mechanics of walking.

The work cited above indicates several necessary requirements for the next significant advancement in our understanding of normal and pathological human movement. First, we need to capture the kinematics and kinetics of human movement without the constraints of the laboratory or the encumbrance of placing markers on the limb segments. Second, we need to relate the external features of human movement to the internal anatomical structures (e.g. muscle, bone, cartilage and ligaments) to further our knowledge of musculoskeletal function and pathology.

The results presented here demonstrate that markerless motion capture has the potential to achieve a level of accuracy that facilitates the study of the biomechanics of normal and pathological human movement. The errors affecting the accuracy of a markerless motion capture system can be classified into errors due to limitations of the technical equipment and errors due to the shape and/or size of the object or body under investigation. For instance, the accuracy of markerless methods based on visual hulls is dependent on the number of cameras. Configurations with fewer than 8 cameras resulted in volume estimations greatly deviating from original values and fluctuating enormously for different poses and positions across the viewing volume. Visual hulls were not able to capture surface depressions such as eye sockets and lacked accuracy in narrow spaces such as the arm pit and groin regions. However, a human form can be approximated accurately with the appropriate number of cameras for the specific viewing volume. Configurations with 8 and more cameras provided good volume estimations and consistent results for different poses and positions across the viewing volume. Thus, one multi-camera system can be used for both capturing human shape and human movement.

The work presented here systematically points out that choosing appropriate technical equipment and approaches for accurate markerless motion capture is critical. The processing modules used in this study including background separation, visual hull, iterative closest point methods, etc. yielded results that were comparable to a marker-based system for motion at the knee. While additional evaluation of the system is needed, the results demonstrate the feasibility of calculating meaningful joint kinematics from subjects walking without any markers attached to the limb.

The markerless framework introduced in this work can serve as a basis for developing the broader application of markerless motion capture. Each of the modules can be independently evaluated and modified as newer methods become available, thus making markerless tracking a feasible and practical alternative to marker based systems. Markerless motion capture systems offer the promise of expanding the applicability of human movement capture, minimizing patient preparation time, and reducing experimental errors caused by, for instance, inter-observer variability. In addition, gait patterns can not only be visualized using traces of joint angles but sequences of snapshots (Figure 4) can be easily obtained that allow the researcher or clinician to combine the qualitative and quantitative evaluation of a patient's gait pattern. Thus, the implementation of this new technology will allow for simple, time-efficient, and potentially more meaningful assessments of gait in research and clinical practice.
